# Association of family wellbeing with forwarding and verifying COVID-19-related information, and mediation of family communication quality

**DOI:** 10.3389/fpubh.2022.948955

**Published:** 2022-08-17

**Authors:** Bonny Yee-Man Wong, Sai Yin Ho, Shirley Man Man Sit, Wei Jie Gong, Agnes Yuen Kwan Lai, Man Ping Wang, Tai Hing Lam

**Affiliations:** ^1^Department of Health Science, School of Nursing and Health Studies, Hong Kong Metropolitan University, Hong Kong, Hong Kong SAR, China; ^2^School of Public Health, The University of Hong Kong, Hong Kong, Hong Kong SAR, China; ^3^School of Nursing, The University of Hong Kong, Hong Kong, Hong Kong SAR, China

**Keywords:** COVID-19, information sharing, fact-check, information overload, misinformation, family wellbeing

## Abstract

**Objective:**

We assessed the associations of family wellbeing with verifying and subsequently forwarding COVID-19-related information to family members and the mediating effect of the quality of family communication on these associations among Chinese adults in Hong Kong.

**Methods:**

Under the Jockey Club SMART Family-Link Project, we conducted an online population-based survey, using Family wellbeing Scale and questions related to the family communication quality and forwarding and verifying COVID-19 information. Data were collected from 4,891 adults in May 2020. Prevalence estimates of forwarding and verifying COVID-19 information were weighted by sex, age, and education of the general population, and their associations with family wellbeing (ranged 0–10) were analyzed using generalized linear models with mutual adjustment. Their interactive effects on family wellbeing and the mediating effects of family communication quality were examined.

**Results:**

In total, 53.9% of respondents usually/always forwarded COVID-19 information related to their family, 68.7% usually/always verified it before forwarding, and 40.9% did both. Greater family wellbeing was associated with usually/always forwarding [adjusted β (95% CI): 0.82 (0.72–0.92)] and usually/always verifying [0.43 (0.32–0.55)] (both *P* < 0.001) the information. Forwarding and verifying such information showed an additive effect on family wellbeing [1.25 (1.11–1.40)]. Family communication quality mediated the associations of family wellbeing with forwarding (83.7%) and verifying (86.6%) COVID-19-related information.

**Conclusion:**

Forwarding COVID-19 information to family, verifying such information, and especially doing both, were associated with greater family wellbeing, being strongly mediated by the quality of family communication. Individuals should be encouraged to verify COVID-19-related information before forwarding it to family members amidst the COVID-19 pandemic.

## Introduction

Family communication, namely, sharing of information, knowledge, values, and beliefs, is essential for maintaining family relationships and fostering the wellbeing of the entire family and of each family member (1–4). Sharing information with family members and forming family groups on instant messaging applications were found to improve the quality of family communication and enhance wellbeing, both before and during the COVID-19 pandemic (1, 2, 5, 6). The pandemic has caused severe stress, uncertainties, and social isolation, amplifying the need to feel safe and socially connected. Sharing information with family may reduce loneliness and serve as an important source of health-related information (7, 8). We previously reported that individuals who shared COVID-19-related information with family reported greater family wellbeing (9). With technology advancing, the forwarding of information using electronic communication technologies, namely, instant messaging and social media, has become an increasingly prevalent and common behavior (10). People can massively redirect forwarded messages to others, with or without the recipient's consent; however, the recipient may find such messages overwhelming and irrelevant. The effect of forwarding such messages has not been studied so far.

The overabundance of information during the pandemic—also known as an infodemic—has made it difficult for people to find trustworthy sources and reliable guidance when needed (11). The forwarding of COVID-19-related information has led to widespread misinformation on social media that is not backed by the scientific consensus (12–18). Exposure to less trusted information sources (e.g., social media) and misinformation may increase confusion and perceived risks toward COVID-19 (19) and cause psychological distress (20–23), which may eventually lead to conflicts in the family (24, 25).

Verifying (fact checking) information and not sharing COVID-19-related misinformation can help curb the infodemic (19, 26). Forwarding trustworthy COVID-19-related information may promote family wellbeing; in contrast, forwarding unverified information may amplify the infodemic, hampering mental health. The reasons behind sharing unverified information, namely, perceived COVID-19 severity and vulnerability (27), fear and health anxiety (28, 29), importance of messages (30), entertainment, ignorance (e.g., lack of awareness), altruism (31), and coping with information overload (29), were increasingly studied. However, the effects of forwarding unverified COVID-19-related information on family wellbeing remain unclear. We performed a PubMed searched using the keywords “COVID-19,” “family wellbeing,” “information sharing,” “forwarding information,” “verifying information,” “fact check,” “information overload” and “misinformation” up to April 2022. We found that only one survey conducted prior to the current study and reported the association between the implementation of COVID-19 preventive measures and family wellbeing and the minor mediating effect of sharing COVID-19-related information with family in the association of individual health literacy and preventive measures (32).

To date, no report has examined how the handling of COVID-19 information affects family wellbeing. However, it is crucial to explore how the handling of COVID-19-related information [e.g., verifying the information and then forwarding it to family, which is highly recommended to confront the infodemic (19, 26)] is associated with family wellbeing to provide insights for future research and to determine best practices on strategies to protect family wellbeing from the infodemic.

Given the high Internet (91.7%) and social media (98.0%) penetration rates (33) and the high prevalence of using the Internet to search for information (95.9%) and to communicate (98.9%) (34) in Hong Kong, this study aimed to examine (1) the independent associations of forwarding and verifying COVID-19-related information with family wellbeing, (2) the interaction between forwarding and verifying such information on family wellbeing, and (3) the mediating effect of the quality of family communication in such associations among Chinese adults in Hong Kong.

## Materials and methods

### Study design and participants

The present population-based survey study, known as the first Family Amidst COVID-19 (FamCov1) survey and conducted under the Jockey Club SMART Family-Link Project, was performed in Hong Kong between 26 and 31 May 2020. The study aimed to recruit as many respondents as possible within these 6 days as wave 2 of the COVID-19 outbreak was under control during this period. The eligibility criteria were as follows: (1) adults in Hong Kong aged 18 years or older and (2) able to read and understand Traditional Chinese. Details of the conducted survey have been published in previous reports (6, 35, 36). In short, a probability- and non-probability-based online panels were invited to complete a self-administered online survey *via* email through the Hong Kong Public Opinion Research Institute, a well-known local survey agency (37). A total of 20,103 invitation emails were opened; 6,956 individuals accessed the survey link, of which 4,921 shared useable data after providing informed consent (response rate, 24.5%). After excluding 30 respondents who had no family members, a total of 4,891 respondents were included in the current study. The study was approved by the Institutional Review Board of the University of Hong Kong/Hospital Authority Hong Kong West Cluster (UW 20-238).

### Measurements

#### Independent variables

Forwarding COVID-19 information to family members refers to a specific information sharing behavior on digital platforms such as social media (e.g., Facebook) and instant messaging applications (e.g., WhatsApp). We asked: “When the pandemic was severe, how often did you forward COVID-19-related information to your family members?” In terms of verification, the following question was asked: “When the pandemic was severe, how often did you verify before forwarding COVID-19-related information to family members?” (38). The responses to both questions were recorded as 0 (never) to 5 (50–50) to 10 (always) or “I don't know/refuse to answer,” which were recoded into binary variables [seldom/sometimes (0–6) and usually/always (7–10)] with “do not know/refuse to answer” considered as missing data.

#### Dependent variables

Family wellbeing was measured with the Family wellbeing Scale, which was developed based on the Chinese adults' perspectives on family wellbeing in Hong Kong, and included three questions related to perceived family health, happiness, and harmony (4). The three questions, which also were used in our previous studies (1, 5, 32, 36), were “Do you think your family is (1) healthy, (2) happy, and (3) harmonious?” The responses were scored from 0 to 10 (0 = very unhealthy/unhappy/inharmonious; 5 = 50–50; 10 = very healthy/happy/harmonious). The sum of the three item scores divided by 3 was the composite score of family wellbeing (2, 32). The quality of family communication was assessed using the question “How good do you find the quality of communication between you and your family members?,” with responses scored from 0 to 10 (0 = very bad; 5 = 50–50; 10 = very good) (6).

#### Covariates

Data related to the sex, age group, education, household monthly income, number of cohabitants, and housing status of the respondents were collected (35, 36, 39). Sociodemographic variables were recoded: age (18–24, 25–44, 45–54, and 65 years or older), household income [less than or equal to the median monthly household income per person in Hong Kong (low) (40) vs. high], education (secondary or below vs. postsecondary), and housing (rented vs. owned). A socioeconomic score (SES; range 0–3) was obtained by summing the scores of education (0 = secondary education or lower and 1 = post-secondary education), household income (0 = low and 1 = high), and housing status (0 = rented and 1 = owned). The SES was further recoded as low (0–1), medium (2), or high (3) according to similar characteristics relating SES scores of 0 and 1 (35, 36, 39).

### Statistical analysis

The prevalence estimates of forwarding and verifying COVID-19-related information were weighted by the sex, age, and education of the Hong Kong general population (41, 42). The independent samples *t*-test and one-way ANOVA were used to compare the quality of family communication and family wellbeing based on the respondents' characteristics and behaviors of forwarding and verifying COVID-19-related information. The magnitude of the differences was demonstrated using the effect size (ES): eta-squared (η^2^) for variables with two or more groups and Cohen's *d* for binary variables. Generalized linear models were used to calculate the adjusted odds ratio (OR) and 95% CI of the quality of family communication and family wellbeing for behaviors of verifying and forwarding COVID-19-related information to examine independent associations, adjusting for each other and socio-demographic characteristics. Cross-product terms of verifying and forwarding COVID-19-related information were added in the regression models to examine the interactions. However, no significant interaction was found, and the additive effects of forwarding and verifying COVID-19-related information on family wellbeing were further examined. A composite variable was created by combining the forwarding and verification of COVID-19-related information into four groups: (1) both seldom/sometimes, (2) usually/always forwarding and sometimes/seldom verifying, (3) usually/always verifying and seldom/sometimes forwarding, and (4) both usually/always. The association of the created variable with family wellbeing was then tested using the generalized linear model, adjusted for sociodemographic factors. *PROCESS Macro v3.5* by Hayes, a well-known mediation analysis tool in *IBM SPSS*, was used to examine the mediating (indirect) effect of the quality of family communication on the associations of forwarding and verifying COVID-19-related information with family wellbeing (43, 44). Bias-corrected bootstrap CI method with 5,000 replications was used to obtain the 95% CIs of the direct and indirect effects of verifying and forwarding COVID-19-related information on family wellbeing mediated *via* the quality of family communication, with adjustment for verifying in the analysis of forwarding (and vice versa) and sociodemographic factors. *P* < 0.05 was considered statistically significant. To test the robustness of results, the analyses were repeated with re-categorization of forwarding and verification of COVID-19-related information [less than half the time (score < 5) vs. half the time or more (score ≥ 5)]. All the data were analyzed using IBM SPSS v26.

## Results

Table 1 shows that 50.1 and 41.2% of the respondents were aged 25–44 and 45–64 years, respectively, and 43.7% were man. After weighting, 53.9% of the respondents were found to usually/always forward COVID-19-related information to family members, whereas 68.7% usually/always verified the information before forwarding. Those who usually/always forwarded such information reported better quality of family communication (mean ± SD, 7.04 ± 1.68 vs. 5.78 ± 2.19; *P* < 0.001; ES: 0.65) and greater family wellbeing (7.45 ± 1.43 vs. 6.49 ± 1.83; *P* < 0.001; ES, 0.59) than those who seldom/sometimes did so. Those who usually/always verified such information also reported better quality of family communication (6.62 ± 1.98 vs. 5.97 ± 2.14; *P* < 0.001; ES, 0.32) and greater family wellbeing (7.14 ± 1.65 vs. 6.61 ± 1.78; *P* < 0.001; ES, 0.31). With the variables of forwarding and verifying COVID-19-related information combined, 18.0% of respondents seldom/sometimes did both and 40.9% usually/always did both; family wellbeing was the greatest among those who usually/always did both and the least among those who seldom/sometimes did both (7.52 ± 1.44 vs. 6.25 ± 1.90; *P* < 0.001; ES, 0.09).

**Table 1:** Sociodemographic characteristics and behaviors of forwarding and verifying COVID-19 information (*N* = 4,891).

	**Unweighted**		**Weighted**			**Family communication**				**Family wellbeing**			
	***n***	**(%)**	***n***	**(%)**	**Effect size^∧^**	**Mean**	**(SD)**	***P*** ^*^	**Effect size^∧∧^**	**Mean**	**(SD)**	***P*** ^*^	**Effect size^∧∧^**
**Sex**													
Male	2,138	(43.7)	2,295	(47.1)	0.03	6.45	(2.08)	0.89	0.004	7.05	(1.72)	0.098	0.05
Female	2,753	(56.3)	2,583	(52.9)		6.44	(2.02)			6.97	(1.69)		
**Age group, years**													
18–24	219	(4.5)	416	(8.5)	0.29	5.31	(2.44)	<0.001^#^	0.05	6.01	(2.23)	<0.001^#^	0.05
25–44	2,449	(50.1)	1,581	(32.4)		6.11	(2.13)			6.77	(1.75)		
45–64	2,013	(41.2)	1,839	(37.7)		6.88	(1.80)			7.32	(1.53)		
65 or above	210	(4.3)	1,041	(21.3)		7.31	(1.46)			7.69	(1.25)		
							***P*** **for trend**	<0.001			***P*** **for trend**	<0.001	
**Education**													
Secondary or below	659	(13.6)	3,183	(65.7)	0.53	6.75	(1.84)	<0.001	0.18	7.20	(1.48)	<0.001	0.14
Postsecondary	4,199	(86.4)	1,662	(24.3)		6.39	(2.07)			6.97	(1.73)		
**Household monthly income**													
Lower	1,270	(29.8)	2,201	(52.6)	0.23	6.13	(2.11)	<0.001	0.22	6.69	(1.82)	<0.001	0.27
Higher	2,986	(70.2)	1,986	(47.4)		6.58	(1.99)			7.15	(1.62)		
Housing													
Rent	1,603	(33.9)	1,744	(36.6)	0.03	6.18	(2.10)	<0.001	0.21	6.73	(1.76)	<0.001	0.25
Owned	3,120	(66.1)	3,025	(63.4)		6.61	(2.00)			7.16	(1.65)		
**Socioeconomic score**													
Low (0–1)	790	(18.9)	2,160	(52.3)	0.40	6.17	(2.07)	<0.001^#^	0.007	6.73	(1.76)	<0.001^#^	0.01
Middle (2)	1,497	(35.8)	1,376	(33.3)		6.41	(2.03)			6.93	(1.73)		
High (3)	1,891	(45.3)	595	(14.4)		6.64	(2.01)			7.23	(1.62)		
							***P*** **for trend**	<0.001			***P*** **for trend**	<0.001	
**Forwarding COVID-19 information**													
Seldom/sometimes	2,304	(47.3)	2,238	(46.1)	0.01	5.78	(2.19)	<0.001	0.65	6.49	(1.83)	<0.001	0.59
Usually/always	2,569	(52.7)	2,615	(53.9)		7.04	(1.68)			7.45	(1.43)		
**Verifying COVID-19 information**													
Seldom/sometimes	1,297	(26.1)	1,506	(31.3)	0.052	5.97	(2.14)	<0.001	0.32	6.61	(1.78)	<0.001	0.31
Usually/always	3,583	(73.9)	3,310	(68.7)		6.62	(1.98)			7.14	(1.65)		
**Always forwarding and verifying**													
Neither	774	(16.0)	869	(18.0)	0.06	5.48	(2.27)	<0.001^#^	0.10	6.25	(1.90)	<0.001^#^	0.09
Usually/always forwarding	493	(10.2)	638	(13.2)		6.73	(1.66)			7.18	(1.38)		
Usually/always verifying	1,509	(31.1)	1,339	(27.8)		5.93	(2.12)			6.61	(1.78)		
Both	2,074	(42.8)	1,972	(40.9)		7.12	(1.69)			7.52	(1.44)		
							***P*** **for trend**	<0.001			***P*** **for trend**	<0.001	
	**Mean**	**(SD)**	**Mean**	**(SD)**	**ES**								
Family communication	7.00	(1.70)	7.12	(1.62)	0.07								
Family wellbeing	6.44	(2.05)	6.62	(1.96)	0.09								

Missing data were excluded.

Socioeconomic score (SES): a composite score of education, household monthly income per person, and housing analyzed as low (0–1), middle (2), and high (3).

Always forwarding and verifying: A composite variable by combining forwarding and verifying COVID-19-related information into four groups: (1) neither (both seldom/sometimes forwarding and verifying), (2) always forwarding (and sometimes/seldom verifying), (3) always verifying (and seldom/sometimes forwarding), and (4) both (always forwarding and verifying).

∧ Effect size (ES) for difference between weighted and unweighted sample: Categorical variables: Cramer's V: 0.10–0.30, small; 0.30–0.50, medium; ≥0.50, large; Continuous variables: Cohen's d: 0.2 (small), 0.5 (medium), 0.8 (large).

∧∧ Effect size (ES) for variables with two or more groups: Eta-squared (η^2^): 0.01 (small), 0.06 (medium), and 0.14 (large); ES for variables with two groups: Cohen's d: 0.2 (small), 0.5 (medium), and 0.8 (large).

* Independent sample t-tests and One-Way ANOVA were performed with unweighted sample.

# Post-hoc analyses showed significant difference between all the groups.

Table 2 shows that compared with seldom/sometimes forwarding COVID-19-related information to family members, usually/always forwarding the information was associated with better quality of family communication, independent of whether the information was verified [adjusted β (95% CI), 1.04 (0.93–1.16); *P* < 0.001]. Similarly, compared with the respondents who seldom/sometimes verified COVID-19-related information, those who usually/always did so reported better quality of family communication [0.51 (0.38–0.65); *P* < 0.001]. Greater family wellbeing was associated with usually/always forwarding [0.82 (0.72–0.92); *P* < 0.001) and verifying [0.43 (0.32–0.55); *P* < 0.001] COVID-19-related information after mutual adjustment. No interaction between forwarding and verifying COVID-19-related information was found (*P* = 0.85); however, compared with those who neither usually/always forwarded nor verified the information, those who usually/always forwarded only [0.82 (0.63–1.01); *P* < 0.001], usually/always verified only [0.43 (0.29–0.58); *P* < 0.001] and usually/always did both [1.25 (1.11–1.40); *P* < 0.001] reported greater family wellbeing.

**Table 2:** Associations of family wellbeing with forwarding and verifying COVID-19 information (*N* = 4891).

	**Family communication**						**Family wellbeing**					
	**Crude β**	**(95% CI)**	***P***	Adj β^*^	**(95% CI)**	***P***	**Crude β**	**(95% CI)**	***P***	Adj β^*^	**(95% CI)**	***P***
**Forwarding COVID-19**												
**information**												
Seldom/sometimes	0			0			0			0		
Usually/always	1.27	(1.16, 1.38)	<0.001	1.04	(0.93, 1.16)	<0.001	0.97	(0.88, 1.06)	<0.001	0.82	(0.72, 0.92)	<0.001
**Verifying COVID-19**												
**information**												
Seldom/sometimes	0			0			0			0		
Usually/always	0.65	(0.52, 0.78)	<0.001	0.51	(0.38, 0.65)	<0.001	0.53	(0.42, 0.64)	<0.001	0.43	(0.32, 0.55)	<0.001
	**Crude** β	**(95% CI)**	***P***	**Adj** β^**^	**(95% CI)**	***P***	**Crude** β	**(95% CI)**	***P***	**Adj** β^**^	**(95% CI)**	***P***
**Always forwarding and**												
**verifying**												
Neither	0			0			0			0		
Usually/always forwarding	1.25	(1.04, 1.47)	<0.001	1.07	(0.84, 1.30)	<0.001	0.93	(0.75, 1.12)	<0.001	0.82	(0.63, 1.01)	<0.001
Usually/always verifying	0.45	(0.29, 0.62)	<0.001	0.56	(0.35, 0.70)	<0.001	0.36	(0.22, 0.50)	<0.001	0.43	(0.29, 0.58)	<0.001
Both	1.64	(1.48, 1.80)	<0.001	1.56	(1.39, 1.73)	<0.001	1.28	(1.14, 1.41)	<0.001	1.25	(1.11, 1.40)	<0.001

Missing data were excluded.

Data were unweighted.

Always forwarding and verifying: A composite variable by combining forwarding and verifying COVID-19 related information into four groups: (1) neither (both seldom/sometimes forwarding and verifying), (2) always forwarding (& sometimes/seldom verifying), (3) always verifying (& seldom/sometimes forwarding), and (4) both (always forwarding and verifying).

* Adjusted for sex, age, SES score, and verifying and forwarding COVID-19 related information mutually.

** Adjusted for sex, age, SES score.

Table 3 shows that the associations of forwarding and verifying COVID-19-related information with family wellbeing were attenuated after including the quality of family communication as a mediator; 86.6 and 83.7% of the independent total effect of forwarding and verifying COVID-19-related information on family wellbeing was mediated by the quality of family communication, respectively. Moreover, 85.6% of the total effect of forwarding as well as verifying COVID-19-related information on family wellbeing was mediated by the quality of family communication.

**Table 3:** Adjusted indirect, direct, effect of forwarding, and verifying the COVID-19 information on family wellbeing *via* family communication (*N* = 4,891).

		**Family wellbeing**
		**β^∧^(95% CI)**
Forwarding [ Ref: seldom/sometimes (*n* = 2,304)]	Indirect effect (through mediator)	0.71 (0.63, 0.79)^***^
	Direct effect (without mediator)	0.11 (0.05, 0.17)^**^
	Total effect (direct and indirect)	0.82 (0.72, 0.92)^***^
	Proportion of total effect mediated	86.6%
Verifying [ Ref: seldom/sometimes (*n* = 1,297)]	Indirect effect (through mediator)	0.36 (0.26, 0.45)^***^
	Direct effect (without mediator)	0.08 (0.01, 0.14)^*^
	Total effect (direct and indirect)	0.43 (0.32, 0.55)^***^
	Proportion of total effect mediated	83.7%
		**Family wellbeing**
		β^∧∧^**(95% CI)**
Usually/always forwarding and verifying [Ref: Neither (*n* = 774)]	*Indirect effect (through mediator)*	
	Usually/always forwarding (*n* = 493)	0.73 (0.57, 0.89)^***^
	Usually/always verifying (*n* = 1,509)	0.37 (0.22, 0.51)^***^
	Both (*n* = 2,074)	1.07 (0.94, 1.20)^***^
	*Direct effect (without mediator)*	
	Usually/always forwarding (*n* = 493)	0.09 (-0.03, 0.20)
	Usually/always verifying (*n* = 1,509)	0.07 (-0.02, 0.15)
	Both (*n* = 2,074)	0.18 (0.10, 0.27)^***^
	*Total effect (direct and indirect)*	
	Usually/always forwarding (*n* = 493)	0.82 (0.63, 1.01)^***^
	Usually/always verifying (*n* = 1,509)	0.43 (0.29, 0.58)^***^
	Both (*n* = 2,074)	1.25 (1.11, 1.40)^***^
	*Proportion of total effect mediated*	85.6%

Missing data were excluded.

Data were unweighted.

Socioeconomic score: a composite score of education, household monthly income per person, and housing analyzed as low (0–1), middle (2), and high (3).

Always forwarding and verifying: A composite variable by combining forwarding and verifying COVID-19-related information into 4 groups: (1) neither (both seldom/sometimes forwarding and verifying), (2) always forwarding (and sometimes/seldom verifying), (3) always verifying (and seldom/sometimes forwarding), and (4) both (always forwarding and verifying).

∧ Adjusted for sex, age, and SES score.

∧∧ Adjusted for sex, age, SES score and verifying and forwarding COVID-19-related information mutually.

* P < 0.05;

** P < 0.01;

*** P < 0.001.

The results of the robustness analysis are shown in Supplementary table 1; similar results were obtained after re-categorizing the forwarding and verifying of COVID-19-related information. The forwarding of COVID-19 information >50% of the time was associated with greater family wellbeing [2.96 (2.59, 3.33), *P* < 0.001]. The corresponding figure for verifying such information was 1.62 (1.05, 2.19), *P* < 0.001. Similarly, Supplementary table 2 shows that the 91.2% and 82.8% of the total effect of forwarding and verifying COVID-19-related information on family wellbeing was mediated by the quality of family communication.

## Discussion

We have first shown that usually/always forwarding COVID-19-related information to family members, usually/always verifying it before forwarding, and especially doing both were associated with greater family wellbeing and that these associations were significantly mediated by the quality of family communication.

Our results show that forwarding COVID-19-related information to family members was associated with greater family wellbeing, which was mediated by the quality of family communication. The perceived proper use of instant messaging was shown to help overcome the geographical constraints (45) and encourage family communication (46). During the COVID-19 pandemic, with social distancing measures in place, instant messaging has become instrumental in connecting family members (47, 48). Message forwarding is one of the most common core functions of instant messaging applications (10), and forwarding COVID-19-related information can initiate discussions (49) and increase family communication. Through such interactions, family members can support one another to alleviate the impacts of COVID-19 on their mental health (50) and enhance family wellbeing (3). Yet, any family member who is obsessed with COVID-19 may easily forward large numbers of messages to others with or without their consent. Passive recipients of the forwarded messages might find those messages irrelevant or overwhelming (10). Future studies should investigate how family members would respond to forwarded COVID-19-related information.

We found that compared with seldom/sometimes verifying COVID-19-related information, usually/always verifying such information before forwarding it to family members was associated with greater family wellbeing, which was also mediated by the quality of family communication. Verifying before forwarding may reduce the spread of misinformation and circumvent misperceptions related to COVID-19 (51, 52), contradictory information and conflicts (24), and psychological distress (20–23). However, many motives not to verify COVID-19-related information before spreading included perceived herd behavior (willingness to spread the information as many do so) (29), perceived COVID-19 severity and vulnerability (27), fear and health anxiety (28, 29), importance of messages (30), entertainment, ignorance (e.g., lack of awareness), altruism (31), and coping with information overload (29). A mixed-method study reported that Chinese older adults tended to forward unverified health-related information because their main purpose of forwarding the information was to maintain relationships rather than provide real information support (48). However, Chinese people find it challenging to correct a senior relative's forwarded misinformation because their culture emphasizes that elders should be respected (53). To reduce the spread of misinformation and contradictory information and to avoid conflicts, which will help promote family wellbeing, it is important to encourage individuals of all ages to verify COVID-19-related information before forwarding it to family members by addressing their motives. Moreover, future studies need to evaluate the moderating effect of specific verification methods on the association between forwarding COVID-19-related information and family wellbeing as we did not ask how the respondents verified COVID-19 information to evaluate its appropriateness and effects on family wellbeing.

The overall effects of forwarding COVID-19-related information on family wellbeing were greater than those of verifying such information (adjusted β: 0.82 vs. 0.43). We assumed that more frequent verification would lead to more accurate information, but only if appropriate sources were used. In addition to the frequency of information verification, eHealth literacy and verification sources are important factors associated with the accuracy of COVID-19-related information (7, 30, 54, 55). Future studies should confirm and compare the strengths of these associations and examine how sources of information verification and eHealth literacy are related to the quality of family communication and family wellbeing.

We did not observe any interaction between forwarding and verifying COVID-19-related information on family wellbeing. However, the additive effects (usually/always forwarding and verifying) resulted in the highest scores of family wellbeing, which were also greatly mediated by the quality of family communication. Whether the associations were causal warrants further studies.

Although the positive association of family wellbeing with forwarding and verifying COVID-19 information mediated by the quality of family communication, nearly 25% of the respondents seldom/sometimes forwarded as well as verified the information and only 40% usually/always did both. Therefore, there is an urge to advocate the importance of verifying and forwarding COVID-19 information to family in enhancing family communication and wellbeing during the COVID-19 pandemic. A moderate level of fear of COVID-19 in Hong Kong adults exits and ≈40% of them perceive COVID-19-related harms to their family (35, 36). Education, social, and health professionals should thus encourage people to verify and forward reliable COVID-19-related information to family to promote family communication and wellbeing during and after COVID-19 pandemic. Moreover, verifying COVID-19-related information from the Internet is challenging but essential to combat the infodemic; public health professionals should educate people about basic digital literacy (e.g., cross-checking different information sources, visiting reliable sources, visiting the actual source instead of a website summary) to increase the ability of information verification (56, 57).

Our study had several limitations. First, all data were self-reported and subject to recall errors. Second, although the temporality of the associations could not be ascertained, the forwarding and verifying of COVID-19-related information was considered at an earlier time-point (during wave 2 of the pandemic) as compared with family communication and wellbeing (after wave 2). Thus, prospective studies are required to ascertain the associations noted in this study. Third, social desirability cannot be avoided in self-administered questionnaires; however, the respondents were recruited *via* email to complete the self-administered, anonymous online questionnaire, which could reduce social desirability in reporting forwarding and verifying COVID-19-related information (58, 59). Fourth, our sample had more educated respondents than the general population. Thus, the prevalence estimates, even after weighting, might not be generalizable to the general population. The educated group may be more digitally health literate (60) and thus more aware of the importance of information verification (30). However, only slight differences were found in the behaviors of forwarding and verifying COVID-19-related information and the family wellbeing between the unweighted and weighted samples.

## Conclusions

We have first shown the association of family wellbeing with verifying and then forwarding COVID-19-related information to family members as well as a strong mediating effect of the quality of family communication. However, prospective studies are warranted to confirm the observed associations. Considering that the COVID-19 pandemic is still underway and causing stress and uncertainties with detrimental effects on families, public healthcare professionals should encourage the verification and forwarding of COVID-19-related information to family members to ensure family communication and wellbeing.

## Data availability statement

The raw data supporting the conclusions of this article will be made available by the authors, without undue reservation.

## Ethics statement

The studies involving human participants were reviewed and approved by Institutional Review Board of the University of Hong Kong/Hospital Authority Hong Kong West Cluster & Ref. no: UW 20-238. The patients/participants provided their written informed consent to participate in this study.

## Author contributions

BW: Conceptualisation, Data curation, project administration, formal analysis and writing—original draft. SS and WG: Writing—review and editing. AL: Conceptualization and writing—review and editing. SH, MW, and TL: Supervision, conceptualization, and writing—review and editing. All the authors participated in the critical review of this study and provided final approval for the publication submission.

## Funding

This research was funded by the Hong Kong Jockey Club Charities Trust.

## Conflict of interest

The authors declare that the research was conducted in the absence of any commercial or financial relationships that could be construed as a potential conflict of interest.

## Publisher's note

All claims expressed in this article are solely those of the authors and do not necessarily represent those of their affiliated organizations, or those of the publisher, the editors and the reviewers. Any product that may be evaluated in this article, or claim that may be made by its manufacturer, is not guaranteed or endorsed by the publisher.
