# Erratum: Association of family wellbeing with forwarding and verifying COVID-19-related information, and mediation of family communication quality

**DOI:** 10.3389/fpubh.2022.1040606

**Published:** 2022-11-10

**Authors:** Frontiers Production Office

**Affiliations:** Frontiers Media SA, Lausanne, Switzerland

**Keywords:** COVID-19, information sharing, fact-check, information overload, misinformation, family wellbeing

Due to a production error, author Bonny Yee-Man Wong was referenced incorrectly in the citation.

In the original version, they were incorrectly written as Wong B-M. The correct version is Wong BY.

The publisher apologizes for this mistake. The original version of this article has been updated.

