# Supplementary material for: Association of family wellbeing with forwarding and verifying COVID-19-related information, and mediation of family communication quality
Source: Front Public Health. 2022 Aug 17;10:948955. doi: 10.3389/fpubh.2022.948955 (PMC9428309; doi:10.3389/fpubh.2022.948955)
Supplement: Supplementary file 1 [file Data_Sheet_1.docx]

**Supplementary Table 1. Sensitivity analysis: Associations of family well-being with forwarding and verifying COVID-19 information (N=4891)**

|  |  | **Family communication** | | | |  | |  |  |  | **Family well-being** | | | |  |  |  |  |
| --- | --- | --- | --- | --- | --- | --- | --- | --- | --- | --- | --- | --- | --- | --- | --- | --- | --- | --- |
|  | **n (%)** | **Crude β** | **(95% CI)** | | **P** | | **Adj β ^*^** | **(95% CI)** | | **P** | **Crude β** | **(95% CI)** | | **P** | **Adj β ^*^** | **(95% CI)** | | **P** |
|  |  |  |  |  |  | |  |  |  |  |  |  |  |  |  |  |  |  |
| **Forwarding COVID-19 information** |  |  |  |  |  | |  |  |  |  |  |  |  |  |  |  |  |  |
| Less than half the time (score < 5) | 1089 (22.3) | 0 |  |  |  | | 0 |  |  |  | 0 |  |  |  | 0 |  |  |  |
| Half the time or more (score ≥ 5) | 3784 (77.7) | 1.60 | (1.47, | 1.73) | <0.001 | | 1.32 | (1.18, | 1.47) | <0.001 | 3.56 | (3.23, | 3.89) | <0.001 | 2.96 | (2.59, | 3.33) | <0.001 |
| **Verifying COVID-19 information** |  |  |  |  |  | |  |  |  |  |  |  |  |  |  |  |  |  |
| Less than half the time (score < 5) | 383 (7.9) | 0 |  |  |  | | 0 |  |  |  | 0 |  |  |  | 0 |  |  |  |
| Half the time or more (score ≥ 5) | 4467 (92.1) | 1.24 | (1.03, | 1.45) | <0.001 | | 0.64 | (0.42, | 0.87) | <0.001 | 2.76 | (2.24, | 3.29) | <0.001 | 1.62 | (1.05, | 2.19) | <0.001 |
|  |  | **Crude β** | **(95% CI)** | | **P** | | **Adj β ^**^** | **(95% CI)** | | **P** | **Crude β** | **(95% CI)** |  | **P** | **Adj β ^**^** | **(95% CI)** | | **P** |
| **Forwarding and verifying***** |  |  |  |  |  | |  |  |  |  |  |  |  |  |  |  |  |  |
| Both less than half the time | 257 (5.3) | 0 |  |  |  | | 0 |  |  |  | 0 |  |  |  | 0 |  |  |  |
| Forwarding: half the time or more | 126 (2.6) | 1.32 | (0.91, | 1.73) | <0.001 | | 1.11 | (0.67, | 1.54) | <0.001 | 2.51 | (1.46, | 3.54) | <0.001 | 2.31 | (1.21, | 3.40) | <0.001 |
| Verifying: half the time or more | 814 (16.8) | 0.43 | (0.16, | 0.70) | <0.001 | | 0.55 | (0.26, | 0.84) | <0.001 | 0.78 | (0.09, | 1.46) | <0.001 | 1.32 | (0.59, | 2.06) | <0.001 |
| Both half the time or more | 3653 (75.3) | 1.95 | (1.71, | 2.19) | <0.001 | | 1.90 | (1.63, | 2.16) | <0.001 | 4.21 | (3.59, | 4.82) | <0.001 | 4.36 | (3.70, | 5.03) | <0.001 |

Missing data were excluded.

Data were unweighted.

^*^Adjusted for sex, age, SES score, and verifying and forwarding COVID-19 related information mutually.

^**^Adjusted for sex, age, SES score.

*******Forwarding and verifying: A composite variable by combining forwarding and verifying COVID-19 related information into 4 groups: 1) both less than half the time (score< 5), 2) forwarding half the time or more (& verifying less than half the time), 3) verifying half the time or more (& forwarding less than half the time), and 4) both half the time or more (score≥ 5).

**Supplementary Table 2. Sensitivity analysis: adjusted indirect, direct, effect of forwarding and verifying the COVID-19 information on family well-being via family communication (N=4891)**

|  |  | **Family well-being** |
| --- | --- | --- |
|  |  | **β^ (95% CI)** |
| **Forwarding (Ref: less than half the time (n=1089))** | Indirect effect (through mediator) | 2.70 (2.34, 3.07)*** |
|  | Direct effect (without mediator) | 0.26 (0.04, 0.49)* |
|  | Total effect (direct and indirect) | 2.96 (2.59, 3.34)*** |
|  | Proportion of total effect mediated | 91.2% |
| **Verifying (Ref: less than half the time (n=383))** | Indirect effect (through mediator) | 1.35 (0.79, 1.93)*** |
|  | Direct effect (without mediator) | 0.29 (-0.04, 0.62) |
|  | Total effect (direct and indirect) | 1.63 (1.06, 2.20)*** |
|  | Proportion of total effect mediated | 82.8% |
|  |  | **Family well-being** |
|  |  | **β ^^ (95% CI)** |
| **Forwarding and verifying (Ref: both less than half of the time (n=257))** | *Indirect effect (through mediator)* |  |
|  | Forwarding: more than half the time (126) | 2.26 (1.26, 3.22)*** |
|  | Verifying: more than half the time (814) | 1.15 (0.36, 1.94)*** |
|  | Both more than half the time (3653) | 3.90 (3.16, 4.68)*** |
|  | *Direct effect (without mediator)* |  |
|  | Forwarding: more than half the time (126) | 0.04 (-0.59, 0.68) |
|  | Verifying: more than half the time (814) | 0.18 (-0.24, 0.61) |
|  | Both more than half the time (3653) | 0.47 (0.09, 0.87)*** |
|  | *Total effect (direct and indirect)* |  |
|  | Forwarding: more than half the time (126) | 2.30 (1.20. 3.40)*** |
|  | Verifying: more than half the time (814) | 1.33 (0.60, 2.07)*** |
|  | Both more than half the time (3653) | 4.37 (3.71, 5.04)*** |
|  | *Proportion of total effect mediated* | 91.4% |

Missing data were excluded.

Data were unweighted.

^ Adjusted for sex, age, SES score.

^^Adjusted for sex, age, SES score and verifying and forwarding COVID-19 related information mutually.

Socioeconomic score: a composite score of education, household monthly income per person, and housing analysed as low (0-1), middle (2) and high (3)

Forwarding and verifying: A composite variable by combining forwarding and verifying COVID-19 related information into 4 groups: 1) both less than half the time (score< 5), 2) forwarding half the time or more (& verifying less than half the time), 3) verifying half the time or more (& forwarding less than half the time), and 4) both half the time or more (score≥ 5).

*P<0.05; **P<0.01; ***P<0.001
